# Mendelian randomization analysis does not reveal a causal influence of smoking on rotator cuff tears

**DOI:** 10.1097/MD.0000000000045212

**Published:** 2025-10-10

**Authors:** Guo-Xu Zhang, Wen-Chao Li, Hai-Yun Chen, Hong-Wei Li

**Affiliations:** a Shanghai University of Traditional Chinese Medicine, Shanghai, China; b Department of Orthopedics, The Seventh People’s Hospital Affiliated to Shanghai University of Traditional Chinese Medicine, Shanghai, China; c Department of Orthopedics, Suzhou TCM Hospital affiliated to Nanjing University of Traditional Chinese Medicine, Suzhou, Jiangsu Province, China; d Department of Orthopaedics, Guangdong Hospital of Traditional Chinese Medicine, Guangzhou, Guangdong Province, China.

**Keywords:** cigarettes per day, Mendelian randomization, rotator cuff tears, smoking, smoking cessation, tobacco

## Abstract

While observational studies suggest smoking as a potential risk factor for rotator cuff tears (RCTs), causal evidence remains inconclusive. This bidirectional Mendelian randomization (MR) study investigates genetic causality between smoking behaviors and RCTs risk. Genetic instruments for smoking initiation, cessation, and cigarettes per day were derived from GSCAN (Sequencing Consortium of Alcohol and Nicotine Use) data. RCTs genome-wide association study (GWAS) summary statistics were obtained from a recent GWAS meta-analysis. Causal effects were evaluated using inverse variance weighted (IVW) fixed/random-effects models, supplemented by MR-Egger, weighted median, and MR-PRESSO methods. Sensitivity analyses assessed heterogeneity and horizontal pleiotropy. No significant causal relationship was found between smoking and RCTs and its subtypes. The sensitivity analysis confirmed the robustness of these results. The large MR analysis indicated that smoking may not be causally associated with a risk of RCTs.

## 1. Introduction

Rotator cuff tears (RCTs) are one of the common causes of shoulder pain and disability, with a surgical incidence of rotator cuff disorders ranging from 12 to 185 per 100,000 persons in all states of the United States.^[1,2]^ In 2000, the annual direct medical costs for shoulder disorders in the United States were estimated at $7 billion.^[3]^ The UK witnessed a tenfold increase in procedures for rotator cuff repair and subacromial decompression between 2004 and 2010.^[3]^ It is estimated that up to 30 percent of the elderly population suffer from RCTs, and prevalence escalates with advancing age.^[4,5]^ The area of shoulder pain in patients is usually on the anterolateral or lateral aspect of the shoulder, and the pain may be worse at night, leading to sleep disturbances, or even further disruption of work and leisure, leading to psychosocial problems such as depression and anxiety.^[2–6]^ Therefore, identifying risk factors for RCTs is crucial to enable prevention strategies, facilitate early diagnosis, improve patient quality of life, and mitigate the associated socioeconomic burden on individuals and healthcare systems. Current evidence classifies the primary etiological contributors as either traumatic (e.g., high-energy impacts from motor vehicle collisions or falls exceeding standing height) or degenerative (characterized by age-related tendon attrition, predominantly affecting individuals ≥ 40 years).^[2]^ However, whether smoking is a risk factor for RCTs remains controversial,^[7,8]^ and the majority of studies investigating the association between smoking and RCTs have been based on observational study designs, which are susceptible to confounding (e.g., by age or socioeconomic status) and biases such as reverse causation or behavioral feedback loops (e.g., smoking initiation postinjury as a coping mechanism), thereby limiting causal inference which are susceptible to reverse causation and confounding (e.g., age), limiting the inference of causality.^[9–11]^

Mendelian randomization (MR) is an analytical approach that employs genetic variants robustly associated with exposures as instrumental variables (IV) to assess causal relationships between these exposures and outcomes.^[12]^ This method leverages the principle of independent assortment (Mendel second law), whereby parental alleles are randomly segregated during gamete formation, analogous to the randomization process in controlled trials.^[13]^ As germline genetic variants are fixed at conception and temporally antecedent to disease onset, this design inherently eliminates reverse causation while minimizing confounding by postnatal environmental or behavioral factors.^[14]^ Compared to traditional randomized controlled trials, MR circumvents the substantial time, financial investments, and ethical constraints associated with experimental interventions while maintaining rigorous causal inference standards. In this study, we used single-nucleotide polymorphisms (SNPs) as IV to conduct a two-sample bidirectional MR study to clarify the causal effects between different smoking characteristics and RCTs. We conducted a two-sample MR study using SNPs closely related to smoking as IV to further clarify the causal effect of different smoking characteristics on RCTs.

## 2. Materials and methods

### 2.1. Study design

**Figure 1.**
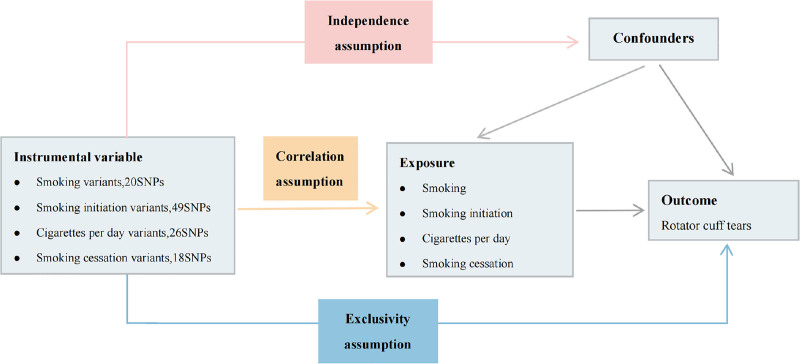
Graphical relationship diagrams of Mendelian randomization. SNPs = single-nucleotide polymorphisms.

The study used a two-sample bidirectional MR design approach to investigate the causal relationship between smoking and RCTs. The MR method requires that the IV satisfy the following 3 assumptions. Assumption I: the assumption of correlation: the genetic variant used as an IV should be strongly correlated with exposure; Assumption II: the assumption of independence: the genetic variant should be unrelated to any confounding factors that may potentially influence exposure and outcome; Assumption III: the assumption of exclusivity: the genetic variant used as an IV should influence outcome only through exposure (Fig. 1).

### 2.2. Data source

To minimize potential confounding bias from ethnic stratification, we limited the population genetic background of the MR study to individuals of European descent. In this study, we pooled data on smoking from the FinnGen consortium, which included 4271 smoking cases and 166,614 controls, as well as data from the GWAS and Sequencing Consortium of Alcohol and Nicotine Use (GSCAN) for 3 different smoking phenotypes. The genome-wide association study (GWAS) on smoking initiation compared current or former regular smokers to never-smokers and involved a population of 1232,091 individuals of European descent. The GWAS on cigarettes per day, which measures the average number of cigarettes smoked daily by current or former smokers, included 337,334 individuals from European populations. The GWAS focusing on smoking cessation compared current smokers to former smokers and included 547,219 individuals from European populations. Pooled statistics on smoking do not include participants from the UK Biobank study. The SNPs associated with RCTs were derived from a GWAS meta-analysis that included 5701 patients and 406,310 controls.^[15]^ The Sources of GWAS summary statistics for the present MR study are presented in Table 1. No further ethics approval was required as the data used in this study were publicly available. Ethical approval and informed consent were obtained in the original studies.

**Table 1:** Characteristics of data in this study.

Dataset	Data source	Sample size (cases)	Number of SNPs	Year	Population
Smoking	FinnGen	170,885 (4271)	21,288,424	2024	European
Smoking initiation	GSCAN	1,232,091	13,933,175	2019	European
Cigarettes per day	GSCAN	337,334	13,863,069	2019	European
Smoking cessation	GSCAN	547,219	14,310,198	2019	European
RCTs	PMID:32663566	412,011 (5701)	16,110,542	2020	European

GSCAN = sequencing consortium of alcohol and nicotine use, PMID = PubMed ID, RCTs = rotator cuff tears.

### 2.3. SNP selection

To meet the correlation hypothesis, we selected SNPs associated with smoking that achieved genome-wide significance (*P* < 5 × 10^−8^) as preliminary instruments. To ensure the independence of IV used for exposure, SNPs in linkage disequilibrium were excluded (r^2^ = 0.001 and kb = 10,000). Since at least 10 eligible IV are required to be included in the MR analysis,^[16,17]^ smoking from the FinnGen consortium, cigarettes per day and smoking cessation GWAS database could only identify 3, 8, and 4 significant and independent SNPs, respectively, when the threshold was set to *P* < 5 × 10^−8^. Therefore, the significance thresholds for smoking from the FinnGen consortium, cigarettes per day, and smoking cessation GWAS were relaxed to *P* < 5 × 10^−6^.^[18,19]^ To meet the independence and exclusion assumptions, we used the LDtrait Tool database to identify and exclude SNPs associated with factors associated with potential confounders (*P* < 5 × 10^−5^), with proposed factors including shoulder impingement syndrome, age > 50 years and arm impedance^[20]^ (https://ldlink.nih.gov/?tab=ldtrait). The *F* statistic was calculated to assess the presence of weak instrumental variable bias. It was computed using the formula *F *= *R*^2^(N − 2)/(1 − *R*^2^), where *R*^2^ represents the ratio of the genetic variance contribution of the exposure factor to the total variation of the exposure factor, and N denotes the sample size for the exposure factor.^[21]^ SNPs with low effect estimates (*F* < 10) were excluded from the analysis. To ensure the correspondence of alleles between exposure and outcome, data were harmonized, and proxies were not used to replace missing SNPs in the outcome data.^[22]^

### 2.4. Statistical analysis

For primary analyses, we employed inverse variance weighting (IVW) to estimate the causal association between smoking and RCTs, as this method provides unbiased estimates with high precision and statistical power when IV exhibit no pleiotropy.^[23,24]^ Heterogeneity across instruments was assessed using Cochran *Q* statistic, with analyses performed via fixed-effects models when *P* > .05 (indicating no substantial heterogeneity) or random-effects models otherwise.^[25]^ In addition, MR-Egger and weighted median were used as complementary methods to MR analysis to estimate causal effects.

The MR-Egger intercept evaluates potential directional pleiotropy; a statistically significant result (*P* < .05) suggests its presence, potentially violating the instrumental variable assumptions of independence and exclusivity.^[14]^ We also applied the MR pleiotropy residual sum and outlier (MR-PRESSO) to detect any possible directional pleiotropy outliers and corrected the IVW estimates by removing outliers.^[26]^ Leave-one-out sensitivity analyses were additionally conducted, sequentially excluding each SNP to further assess the robustness of the findings.^[27]^ MR and sensitivity analysis in the R (version 4.2.2) computing environment using the TwoSampleMR (version 0.6.8) and MR-PRESSO (version 1.0) package.^[14]^

## 3. Results

### 3.1. Causal effects from smoking on RCTs

**Figure 2.**
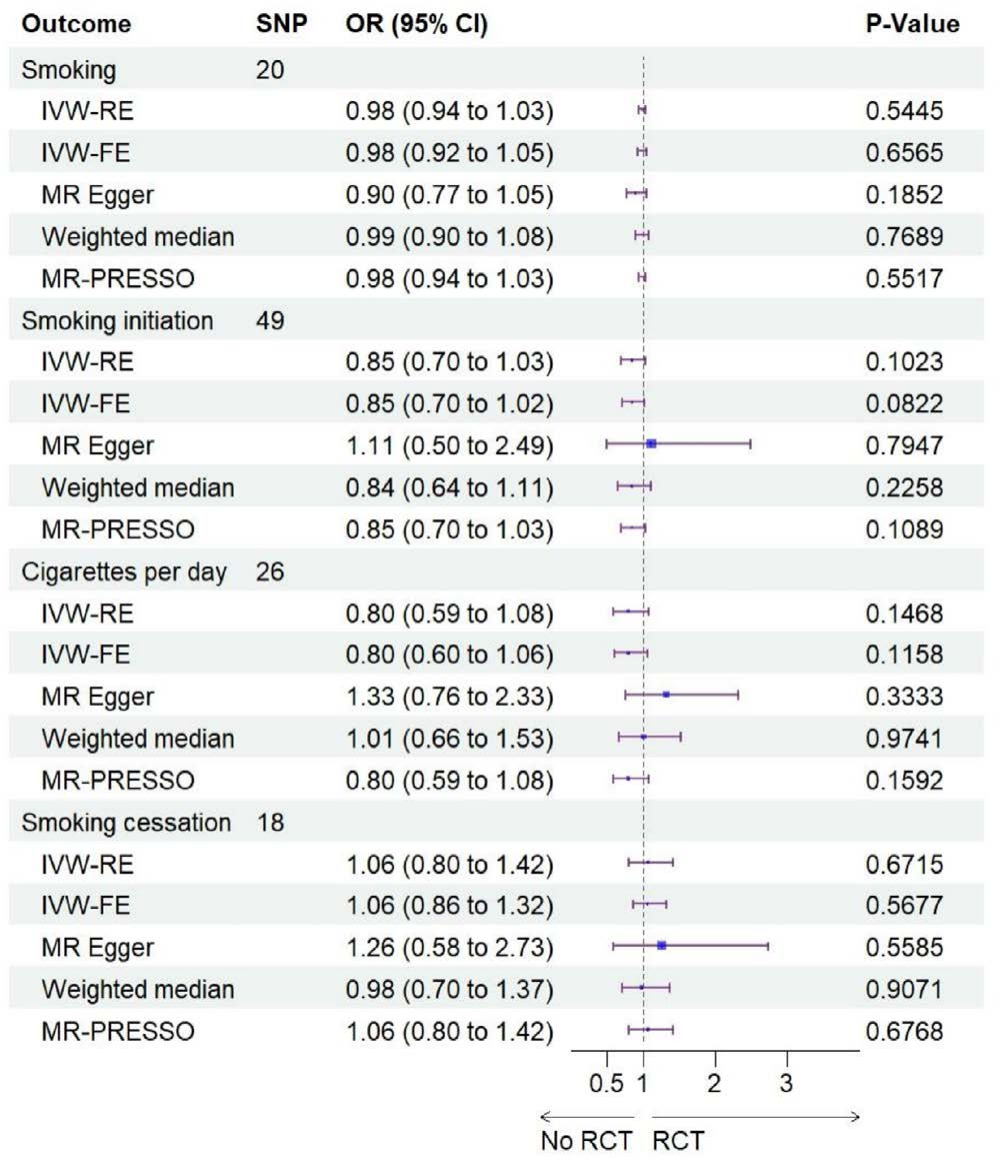
Forest plot of the association of smoking with rotator cuff tears. CI = confidence interval, IVW-FE = inverse-variance weighted fixed-effects, IVW-RE = inverse-variance weighted random-effects, OR = odds ratio.

**Figure 3.**
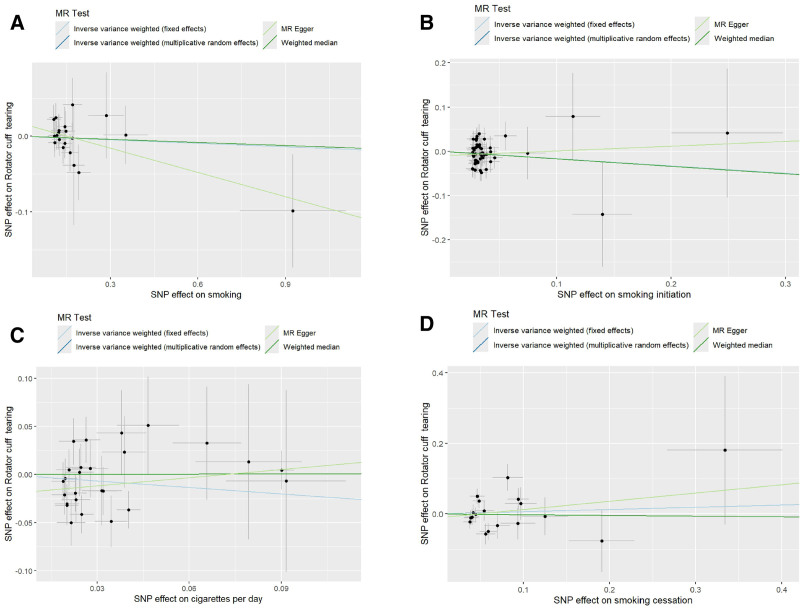
Scatter plot of the Mendelian randomization analysis results between exposures and outcome: (A) smoking on RCTs, (B) smoking initiation on RCTs, (C) cigarettes per day, and RCTs, (D) smoking cessation on RCTs. The slope of each line corresponds to the causal estimate for each method. RCTs = rotator cuff tears.

Following stringent quality control, all SNPs retained as IV exceeded the conventional F-statistic threshold of 10, indicating robust instrument strength (Table S1, Supplemental Digital Content, https://links.lww.com/MD/Q336). These variants were subsequently employed in univariable MR analyses investigating the smoking-rotator cuff tear association. Twenty independent SNPs from the FinnGen consortium were analyzed in MR to assess smoking’s causal relationship with RCTs. The inverse-variance weighted fixed-effects (IVW-FE) method demonstrated no statistically significant causal association ([odds ratio (OR) 0.985, 95% confidence interval (CI) 0.920–1.054; *P* = .6565). Similarly, 49 independent genetic variants were analyzed in a MR study investigating the causal relationship between smoking initiation and RCTs. The IVW-FE method revealed no statistically significant genetic causality (OR = 0.848, 95% CI = 0.705–1.021; *P* = .0822). In the MR framework examining cigarettes per day and RCTs, 26 independent genetic instruments were analyzed. The IVW-FE method revealed no significant genetic association between cigarettes per day and tear risk (OR = 0.799, 95% CI = 0.604–1.057; *P* = .1158). For smoking cessation analyses, 18 genetic variants were employed as instruments, with inverse-variance weighted random-effects (IVW-RE) models similarly demonstrating no causal relationship (OR = 1.065, 95% CI = 0.7964–1.424; *P* = .6715). Other methods, including weighted median, MR-Egger regression, and MR-PRESSO, also verified that there was no significant association between smoking and the risk of RCTs (*P* > .05 for all methods). Detailed results of the MR analyses are shown in Figures 2 and 3.

### 3.2. Sensitivity analysis

**Figure 4.**
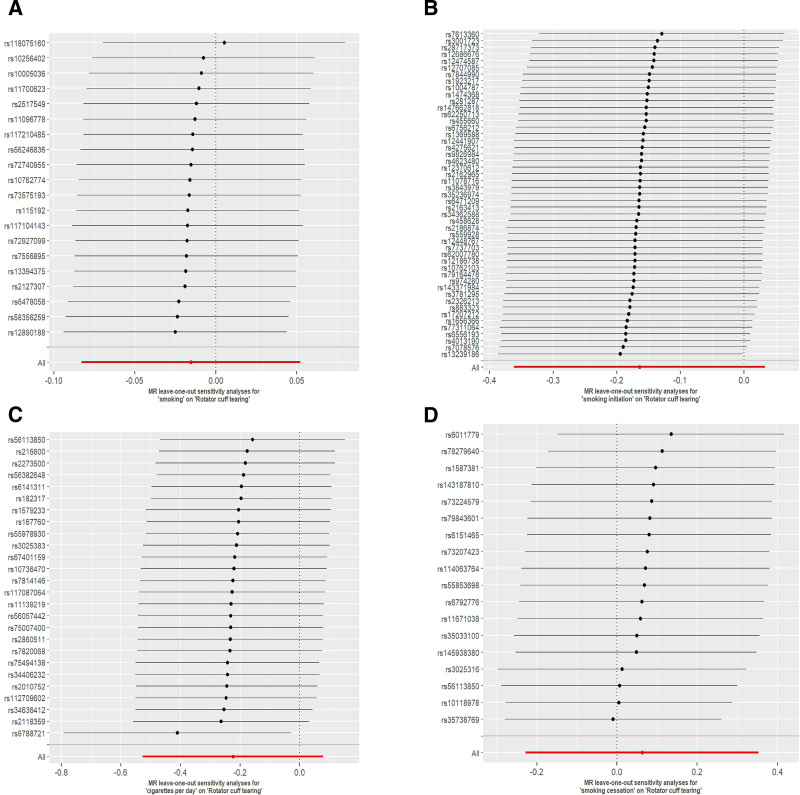
Leave-one-out analysis of the Mendelian randomization results between exposures and outcome: (A) smoking on RCTs, (B) smoking initiation on RCTs, (C) cigarettes per day, and RCTs, (D) smoking cessation on RCTs. RCTs = rotator cuff tears.

Heterogeneity assessments revealed no significant variability in smoking, smoking initiation, or daily cigarette consumption analyses (Cochran Q *P* > .05), whereas smoking cessation analyses demonstrated detectable heterogeneity (*P* < .05). MR-Egger regression identified no evidence of directional pleiotropy across all smoking-related exposures (intercept *P* > .05; Table 2). Funnel plot results showed that the SNPs for each smoking phenotype were largely symmetrical, suggesting stable results (Fig. S1, Supplemental Digital Content, https://links.lww.com/MD/Q335), and the MR-PRESSO test did not find outliers between each of the smoking phenotypes and the risk of RCTs, and the leave-one-out sensitivity test confirmed the robustness of the results (Figure 4).

**Table 2:** Heterogeneity and horizontal pleiotropy assessment for exposure–outcome associations.

Expose	Outcome	Heterogeneity test		MR-Egger pleiotropy test	
		Cochran *Q*	*P*-value	Intercept	*P*-value
Smoking	RCTs	8.496	.970	0.016	.204
Smoking initiation		53.802	.230	−0.009	.497
Cigarettes per day		24.971	.407	−0.020	.051
Smoking cessation		30.435	.016	−0.011	.642
RCTs	Smoking	6.429	.843	0.002	.890
	Smoking initiation	5.218	.876	−0.002	.678
	Cigarettes per day	7.319	.695	−0.002	.622
	Smoking cessation	15.566	.113	−0.001	.950

RCTs = rotator cuff tears.

### 3.3. Causal effects from RCTs on smoking

**Figure 5.**
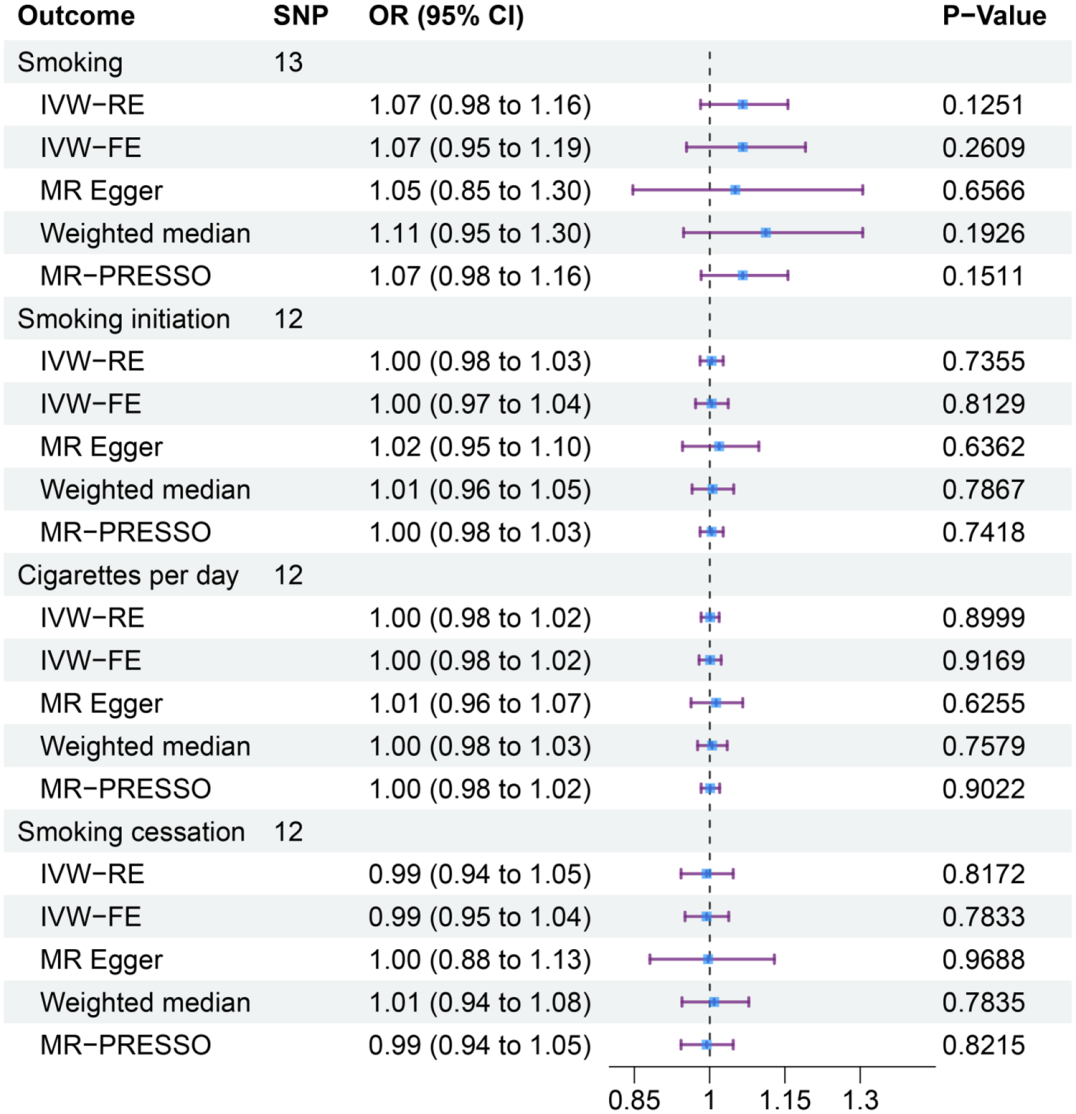
Forest plot of the association of rotator cuff tears with smoking. CI = confidence interval, IVW-FE = inverse-variance weighted fixed-effects, IVW-RE = inverse-variance weighted random-effects, OR = odds ratio.

**Figure 6.**
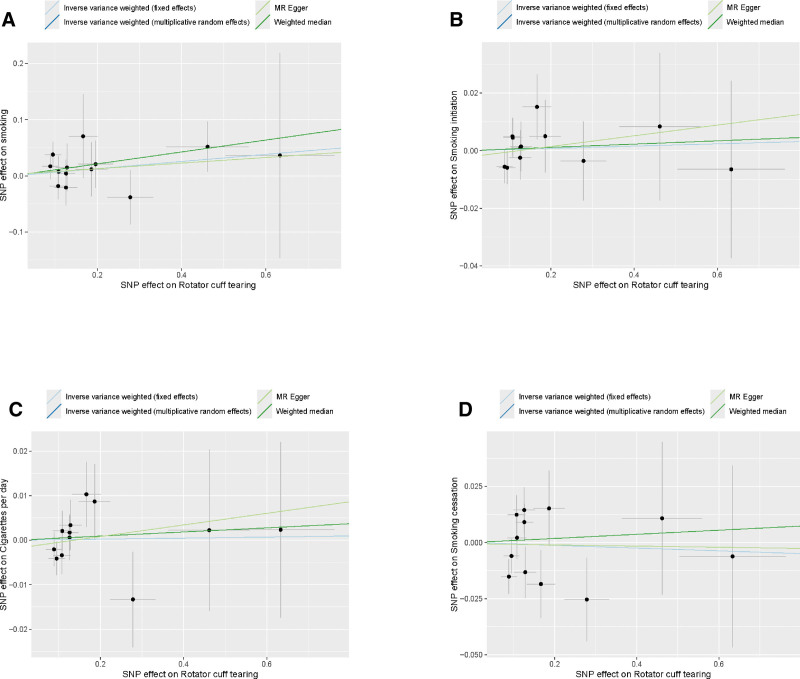
Scatter plot of the Mendelian randomization analysis results between exposures and outcome: (A) RCTs on smoking, (B) RCTs on smoking initiation, (C) RCTs on cigarettes per day, (D) RCTs on smoking cessation. The slope of each line corresponds to the causal estimate for each method. RCTs = rotator cuff tears.

We used the LDtrait Tool database to perform rigorous screening and found that rs4772085 was associated with shoulder impingement syndrome and was excluded. Ultimately, all SNPs strongly associated with RCTs included in the analysis exceeded the conventional *F*-statistic threshold of 10, indicating that the tool is robust (Table S1, Supplemental Digital Content, https://links.lww.com/MD/Q336). Thirteen SNPs were extracted by using RCTs as the exposure variable and smoking as the outcome. The analysis indicated that the IVW-FE method yielded an OR of 1.07 (95% CI = 0.95–1.19; *P* = .2609). Twelve SNPs were extracted by using RCTs as the exposure variable and Smoking initiation as the outcome. The analysis indicated that the IVW-FE method yielded an OR of 1.00 (95% CI = 0.97–1.04; *P* = .8129). In the MR framework examining RCTs and cigarettes per day, 12 independent genetic instruments were analyzed. The analysis indicated that the IVW-FE method yielded an OR of 1.00 (95% CI = 0.98–1.02; *P* = .9169). In the smoking cessation analyses, 12 genetic variants were used as instruments. The results from the IVW-FE models similarly showed no causal relationship, with an OR) of 0.99 (95% CI = 0.95–1.04; *P* = .7833). Other methods, such as the weighted median, MR-Egger regression, and MR-PRESSO, further confirmed that there was no significant association between smoking and the risk of RCTs (*P* > .05 for all methods). The detailed results of the MR analyses are presented in Figures 5 and 6.

**Figure 7.**
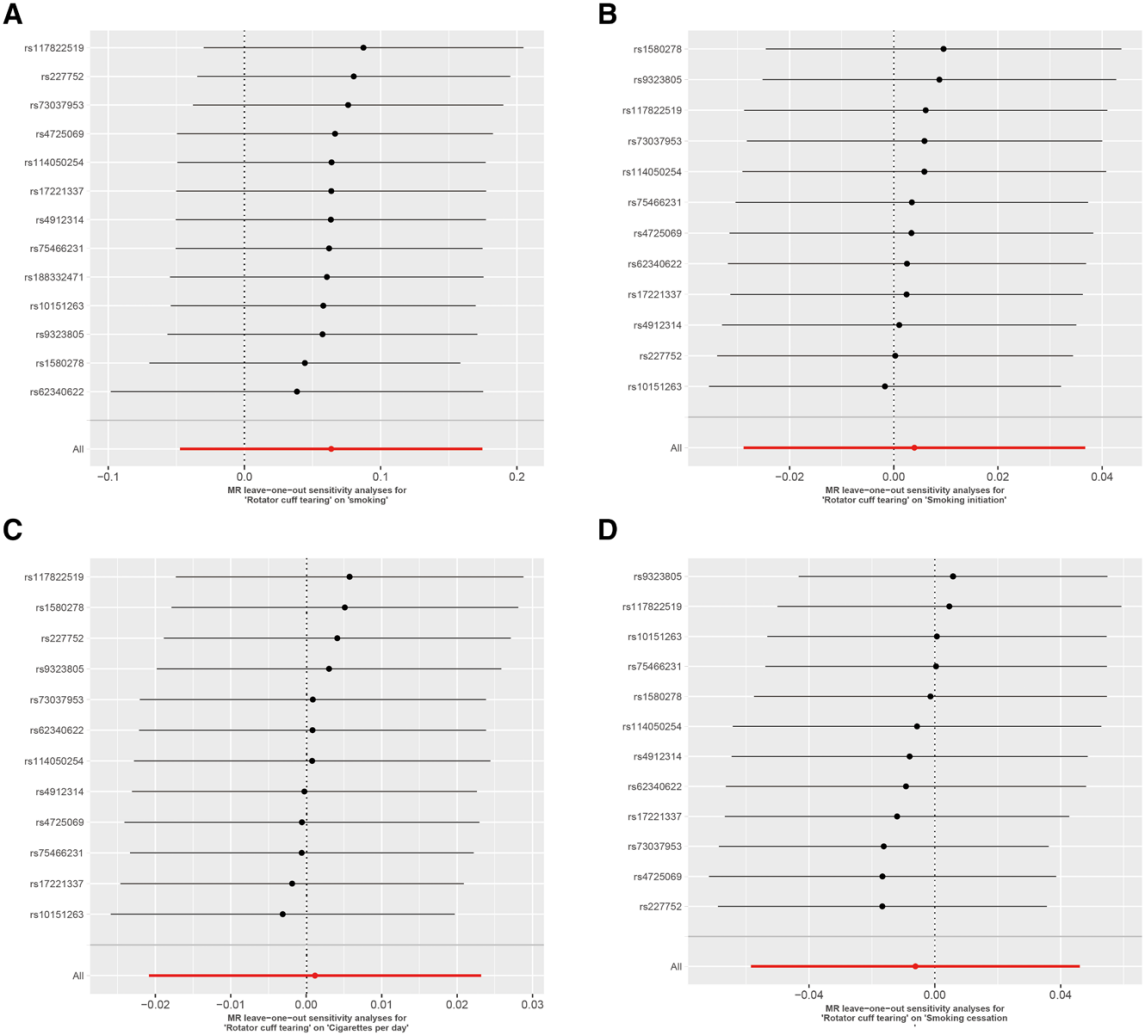
Leave-one-out analysis of the Mendelian randomization results between exposures and outcome: (A) RCTs on smoking, (B) RCTs on smoking initiation, (C) RCTs on cigarettes per day, (D) RCTs on smoking cessation. RCTs = rotator cuff tears.

Heterogeneity assessments showed no significant variability in the analyses of smoking, smoking cessation, smoking initiation, or daily cigarette consumption (Cochran Q *P* > .05). MR-Egger regression found no evidence of directional pleiotropy across all exposures related to RCTs (intercept *P* > .05; Table 2). Funnel plot results indicated that the SNPs were mostly symmetrical, suggesting stable results (Fig. S2, Supplemental Digital Content, https://links.lww.com/MD/Q335). The MR-PRESSO test did not identify any outliers between the RCTs and the risk of each smoking phenotype, and the leave-one-out sensitivity analysis confirmed the robustness of the results (Fig. 7).

## 4. Discussion

To our knowledge, this is the first study to assess the causal relationship between smoking and the risk of RCTs using two-sample bidirectional MR analysis. This study analyzed a large GWAS database to investigate the causal relationship between smoking, smoking initiation, cigarettes per day, smoking cessation, and the risk of RCTs. MR analyses revealed no significant causal associations between these smoking phenotypes and RCTs.

To date, the relationship between smoking and RCTs has not been conclusively established, and the American Academy of Orthopaedic Surgeons (AAOS) Clinical Practice Guideline suggests that future research should focus more on the association between RCTs and factors such as smoking and hypertension and cholesterol.^[4]^ Jeong et al conducted a study analyzing the prevalence of asymptomatic RCTs and their correlates in a Korean cohort comprising 486 participants. They found that approximately 23 individuals (4.7%) exhibited full-thickness RCTs, with smokers demonstrating a significantly higher likelihood of developing full-thickness RCTs compared to nonsmokers (*P *= .002).^[9]^ Furthermore, both a history of smoking and current smoking status were significantly associated with full-thickness RCTs.^[9]^ Lundgreen et al^[28]^ conducted biopsies of ruptured supraspinatus tendons in 25 patients with full-thickness RCTs and demonstrated that the smokers’ group exhibited a significantly lower tendon cell density and a significantly higher apoptotic cell count per square millimeter compared to the nonsmokers’ group. Additionally, smokers displayed more pronounced tendon degeneration.^[28]^ However, the study’s limited sample size and potential confounding due to differing durations of shoulder symptoms between groups may have influenced the trial outcomes.^[28]^ The results of a meta-analysis conducted by Grusky et al.^[8]^ showed that current smokers were twice as likely to develop rotator cuff disorders as nonsmokers, but when the incidence of rotator cuff disorders in never-smokers was compared with that of former smokers and current smokers, no statistically significant difference was found, and the inclusion of this study for a broad range of rotator cuff disorders including rotator cuff tear, rotator cuff syndrome, rotator cuff tendinopathy, rotator cuff tendonitis, etc may have confounded the results.

Although smoking may affect tendon health by reducing microperfusion and tissue oxygenation through nicotine and carbon monoxide, leading to tissue hypoxia, increased apoptosis, and tendon degeneration, MR findings do not support its direct contribution to RCTs.^[29,30]^ Observational studies may overestimate the association between smoking and RCTs, and smoking populations are often accompanied by confounding factors such as occupational heavy labor, low levels of education, or insufficient awareness of exercise protection, whereas MR analyses effectively circumvented such environmental confounders through genetic IV. In a case-control study of 5000 cases of rotator cuff disease, Titchener et al^[31]^ found that 78.9 percent of patients in the case group and 70.5 percent in the control group had a history of smoking and that current smoking was not associated with rotator cuff disease. Previous smoking was associated with the diagnosis of rotator cuff disease, however, this effect disappeared after adjustment for the confounding factor of annual consultation rate.^[31]^ Consistent with the results of this study Zhao et al^[7]^ performed a meta-analysis of 5 studies and showed that smoking did not increase the risk of full-thickness rotator cuff tear (*P* = .09). Han et al^[32]^ used a case-control study to analyze the results of a comparison between 69 patients with RCTs and 51 volunteers, which showed that smoking was not associated with RCTs, whereas measurement of the greater tuberosity humerus angle and greater tuberosity humerus incision angle were independent risk factors for RCTs, and the greater the angle, the more likely that the lateral border of the acromion would friction and impingement would result in RCTs at the lateral site of the bursa, suggesting that mechanical stresses (e.g., repetitive motions) may be a more critical risk factor for RCTs. In 1972 Neer suggested that the rostral shoulder ligament and the anterior third of the acromion impingement were the cause of disability of the shoulder, and subsequently classified the acromion impingement syndrome into 3 phases, and concluded that 95% of rotator cuff injuries progressed from the acromion impingement syndrome.^[33,34]^ Bigliani et al^[35]^ studied 140 cadaveric specimens and classified the morphology of the acromion into 3 types in the supraspinatus exit position, with trisecting hooks accounting for 39% of the cases, and found that 70% of patients with type III acromions had a combined rotator cuff injury. The pathophysiological mechanisms of RCTs are dominated by the accumulation of mechanical loads and degenerative changes, whereas smoking-related metabolic injuries may play a more significant role in disorders of the respiratory and cardiovascular systems rather than in localized tendon tears.^[36,37]^ Some studies have shown that nicotine itself does not negatively affect the musculoskeletal system.^[28,38,39]^

To thoroughly address the potential for reverse causation – a key limitation inherent in observational studies that our MR design aims to circumvent – we further performed reverse MR analyses. This approach explicitly tests the alternative causal direction: whether genetic predisposition to RCTs influences smoking initiation, intensity, or cessation behavior. The rationale is that if RCTs lead to smoking (e.g., as a coping mechanism for pain, disability, or associated psychological distress). We found no evidence that genetic liability for RCTs has any causal effect on any of the smoking phenotypes investigated (Figs. 5 and 6). All ORs were estimated around 1.00 (IVW-FE all *P* > .05), a finding consistently supported by weighted median, MR-Egger, and MR-PRESSO methods. Sensitivity analyses revealed no significant heterogeneity or horizontal pleiotropy, confirming the robustness of these null findings.

The symmetry of our findings – no causality from smoking to RCTs and, crucially, no causality from RCTs to smoking – creates a compelling logical framework. It effectively severs the possibility of a bidirectional causal loop in our genetic analysis. This significantly strengthens our primary conclusion by demonstrating that the null association we observe is not an artifact of reverse causation masking a true effect. Consequently, the positive associations frequently reported in observational literature are more likely attributable to residual confounding by factors such as socioeconomic status, occupational physical demands, or lifestyle factors that correlate with both smoking behavior and the risk of musculoskeletal injury.

This study has several limitations. First, although the exclusivity of the IV was validated through multistep screening and MR-Egger regression, we cannot entirely rule out the possibility that genetic variations influence rotator cuff tear risk via pathways unrelated to smoking (e.g., education, gender, weight, and other factors). Second, the data originated from GWAS summary statistics of European ancestry populations; thus, caution is warranted when extrapolating these findings to other ethnic groups. Moreover, while subtypes of RCTs (e.g., partial vs full tears) may exist, the current GWAS data lack subtype-specific stratification, potentially obscuring differences in genetic effects. Finally, we did not perform advanced bioinformatic analyses such as gene network building or colocalization. While we used the LD trait Tool database, which found no evidence of confounding, these methods could provide deeper insight into the biological pathways linking genetic instruments to the outcome in future studies. Nonetheless, the overall results of this study provide valuable insights into the complex relationship between smoking and RCTs, and it is hoped that more large-sample, high-quality GWAS data, and multicentre, randomized controlled trials will be available in the future to validate and strengthen this finding.

## 5. Conclusion

This is the first MR study to explore the causal relationship between smoking and RCTs, and the results of this study do not provide conclusive evidence to support a direct causal relationship between smoking and the risk of RCTs.

## Acknowledgments

We would appreciate the Sequencing Consortium of Alcohol and Nicotine Use for the GWAS summary statistics of migraine and other GWAS projects, as well as contributing researchers across collaborative GWAS initiatives for data sharing. We further extend our appreciation to the developers of the TwoSampleMR package for their methodological tools enabling robust Mendelian randomization analyses.

## Author contributions

**Data curation:** Guo-Xu Zhang, Hai-Yun Chen, Hong-Wei Li.

**Formal analysis:** Hai-Yun Chen.

**Funding acquisition:** Wen-Chao Li.

**Methodology:** Guo-Xu Zhang, Wen-Chao Li.

**Software:** Guo-Xu Zhang, Wen-Chao Li, Hai-Yun Chen.

**Writing – original draft:** Guo-Xu Zhang.

**Writing – review & editing:** Hong-Wei Li.

## Supplementary Material




