# Supplementary material for: Mendelian randomization analysis does not reveal a causal influence of smoking on rotator cuff tears
Source: Medicine (Baltimore). 2025 Oct 10;104(41):e45212. doi: 10.1097/MD.0000000000045212 (PMC12517924; doi:10.1097/MD.0000000000045212)
Supplement: Supplementary file 1 [file medi-104-e45212-s001.docx]

***Supplementary Material***

**Table S1.** The detailed information of the instrumental variables in each trait

| **Traits** | **SNP** | **EA** | **OA** | **Beta** | **SE** | **P-value** | **F** |
| --- | --- | --- | --- | --- | --- | --- | --- |
| Smoking | rs10256402 | C | T | -0.191613 | 0.041965 | 4.97E-06 | 20.84828826 |
|  | rs10820003 | T | A | 0.117964 | 0.0231863 | 3.63E-07 | 25.88397648 |
|  | rs11096778 | C | T | 0.109032 | 0.0229329 | 1.99E-06 | 22.60398027 |
|  | rs115192 | A | G | 0.12202 | 0.02596 | 2.60E-06 | 22.09263829 |
|  | rs117104143 | C | A | -0.352318 | 0.0767419 | 4.41E-06 | 21.07654821 |
|  | rs117210485 | A | G | -0.174279 | 0.0378054 | 4.03E-06 | 21.25090162 |
|  | rs118075160 | G | A | 0.925297 | 0.182033 | 3.71E-07 | 25.83788567 |
|  | rs12890188 | G | A | -0.112393 | 0.0228672 | 8.88E-07 | 24.15724834 |
|  | rs13394375 | T | C | 0.285194 | 0.0611117 | 3.06E-06 | 21.77844943 |
|  | rs2127307 | A | C | -0.143769 | 0.0283003 | 3.77E-07 | 25.80734588 |
|  | rs4645887 | A | T | -0.124053 | 0.0255402 | 1.19E-06 | 23.59178883 |
|  | rs56246836 | C | T | 0.124278 | 0.0252233 | 8.35E-07 | 24.27613912 |
|  | rs58356259 | T | C | 0.105948 | 0.0229235 | 3.80E-06 | 21.36085286 |
|  | rs67411117 | T | A | -0.148282 | 0.0316642 | 2.83E-06 | 21.92980385 |
|  | rs6849973 | G | C | -0.15791 | 0.0341085 | 3.66E-06 | 21.43329506 |
|  | rs72927099 | C | T | 0.1472 | 0.0312109 | 2.40E-06 | 22.24323513 |
|  | rs7556895 | C | A | 0.123173 | 0.0258175 | 1.83E-06 | 22.76132732 |
|  | rs10005036 | T | G | -0.161916 | 0.0301469 | 7.83E-08 | 28.84623447 |
|  | rs10762774 | A | G | 0.108529 | 0.023012 | 2.40E-06 | 22.24220244 |
|  | rs11700623 | G | A | -0.137642 | 0.0249713 | 3.55E-08 | 30.38187423 |
|  | rs2517549 | A | C | 0.143582 | 0.0253136 | 1.41E-08 | 32.17266876 |
|  | rs6478058 | C | T | 0.170521 | 0.0332949 | 3.03E-07 | 26.2298151 |
|  | rs72740955 | T | C | 0.168848 | 0.0237957 | 1.29E-12 | 50.34887704 |
|  | rs73575193 | T | C | 0.115995 | 0.0242802 | 1.78E-06 | 22.82280061 |
| Smoking cessation | rs79843601 | A | G | -0.0938 | 0.0203 | 3.66E-06 | 21.35069486 |
|  | rs145938380 | T | C | -0.334 | 0.0671 | 6.85E-07 | 24.77686223 |
|  | rs114063764 | A | G | 0.125 | 0.0269 | 3.28E-06 | 21.59304445 |
|  | rs10211384 | G | C | 0.0382 | 0.00811 | 2.52E-06 | 22.18622587 |
|  | rs4675934 | G | A | 0.0742 | 0.0156 | 2.15E-06 | 22.62335584 |
|  | rs6792776 | G | A | 0.0419 | 0.00919 | 4.99E-06 | 20.78717326 |
|  | rs10118978 | A | G | 0.0467 | 0.00859 | 5.43E-08 | 29.55601281 |
|  | rs3025316 | C | T | 0.0941 | 0.0124 | 3.33E-14 | 57.58830409 |
|  | rs143187810 | G | A | 0.191 | 0.0383 | 5.85E-07 | 24.86953123 |
|  | rs73207423 | T | C | 0.0407 | 0.00869 | 2.75E-06 | 21.93550707 |
|  | rs78279640 | C | A | 0.0565 | 0.011 | 2.71E-07 | 26.38213498 |
|  | rs73224579 | G | A | -0.0698 | 0.015 | 3.17E-06 | 21.65343197 |
|  | rs1951389 | C | G | 0.039 | 0.00795 | 9.85E-07 | 24.06541578 |
|  | rs6151465 | T | C | 0.039 | 0.00804 | 1.24E-06 | 23.52965337 |
|  | rs1587381 | A | G | 0.0382 | 0.00767 | 6.27E-07 | 24.80472466 |
|  | rs55853698 | G | T | 0.0447 | 0.00811 | 3.56E-08 | 30.37887945 |
|  | rs56113850 | C | T | -0.0487 | 0.00764 | 1.82E-10 | 40.63213268 |
|  | rs11671038 | G | A | 0.0548 | 0.0111 | 7.75E-07 | 24.37325724 |
|  | rs35033100 | C | T | -0.0973 | 0.0184 | 1.28E-07 | 27.96330163 |
|  | rs6011779 | T | C | -0.0595 | 0.00996 | 2.34E-09 | 35.687298 |
|  | rs35738769 | A | G | 0.0818 | 0.0178 | 4.45E-06 | 21.1185947 |
|  | rs73198625 | T | A | -0.111 | 0.0227 | 1.04E-06 | 23.91072012 |
| Smoking initiation | rs143371984 | T | C | -0.114 | 0.0239 | 2.14E-06 | 22.75166559 |
|  | rs3001723 | A | G | 0.0382 | 0.00626 | 1.07E-09 | 37.2372289 |
|  | rs1923217 | T | C | -0.029 | 0.00567 | 3.04E-07 | 26.15948399 |
|  | rs458628 | A | G | -0.0276 | 0.00591 | 2.98E-06 | 21.80933871 |
|  | rs1004787 | A | G | 0.0307 | 0.00573 | 8.46E-08 | 28.70561145 |
|  | rs6756212 | T | C | -0.0353 | 0.00567 | 5.03E-10 | 38.75989466 |
|  | rs1979004 | A | T | 0.0346 | 0.00601 | 8.66E-09 | 33.143819 |
|  | rs12474587 | T | G | 0.0263 | 0.00574 | 4.54E-06 | 20.99360431 |
|  | rs2107300 | G | C | -0.0406 | 0.00796 | 3.36E-07 | 26.01508365 |
|  | rs2163413 | G | A | -0.0349 | 0.00744 | 2.78E-06 | 22.00413384 |
|  | rs7613360 | T | C | 0.0341 | 0.00584 | 5.33E-09 | 34.09434558 |
|  | rs62250713 | G | A | -0.0289 | 0.00594 | 1.13E-06 | 23.67129897 |
|  | rs9826984 | A | G | -0.0271 | 0.00568 | 1.90E-06 | 22.76361361 |
|  | rs1656366 | A | G | -0.0295 | 0.00596 | 7.45E-07 | 24.4991382 |
|  | rs3843979 | G | T | 0.0331 | 0.00711 | 3.24E-06 | 21.67285279 |
|  | rs77311064 | A | G | 0.0552 | 0.00997 | 3.18E-08 | 30.65399864 |
|  | rs1355334 | A | T | 0.034 | 0.00599 | 1.36E-08 | 32.21836404 |
|  | rs28717373 | T | C | -0.0286 | 0.00583 | 9.79E-07 | 24.06546467 |
|  | rs455660 | C | T | 0.0422 | 0.00808 | 1.75E-07 | 27.27730598 |
|  | rs974280 | T | G | -0.0291 | 0.00591 | 8.10E-07 | 24.24433695 |
|  | rs17207212 | T | C | 0.0367 | 0.00765 | 1.62E-06 | 23.01487144 |
|  | rs7737703 | G | A | 0.0272 | 0.0059 | 3.84E-06 | 21.25362824 |
|  | rs12186738 | T | G | -0.0418 | 0.00785 | 1.06E-07 | 28.35388314 |
|  | rs79164478 | T | C | -0.249 | 0.0487 | 3.05E-07 | 26.14207563 |
|  | rs2162965 | T | C | 0.0365 | 0.00657 | 2.76E-08 | 30.86414743 |
|  | rs883323 | T | C | -0.0325 | 0.00585 | 2.56E-08 | 30.86414743 |
|  | rs6556193 | T | C | 0.0271 | 0.00569 | 1.95E-06 | 22.68367122 |
|  | rs34362588 | T | C | -0.0745 | 0.0162 | 4.43E-06 | 21.14860917 |
|  | rs62447604 | A | G | -0.0646 | 0.0138 | 2.77E-06 | 21.91321795 |
|  | rs1474368 | G | A | -0.0308 | 0.00643 | 1.73E-06 | 22.94450972 |
|  | rs117684381 | A | T | 0.111 | 0.0206 | 8.08E-08 | 29.03426336 |
|  | rs13239186 | T | C | 0.0323 | 0.00613 | 1.35E-07 | 27.76408662 |
|  | rs12707085 | T | C | 0.033 | 0.00702 | 2.56E-06 | 22.09799905 |
|  | rs35236974 | G | A | -0.0385 | 0.00791 | 1.10E-06 | 23.69014872 |
|  | rs2326213 | G | A | -0.0302 | 0.00567 | 1.02E-07 | 28.36919831 |
|  | rs7844990 | C | T | 0.0285 | 0.00581 | 8.98E-07 | 24.06227857 |
|  | rs4623480 | C | T | -0.0344 | 0.00678 | 4.04E-07 | 25.74285986 |
|  | rs7388625 | T | A | -0.0274 | 0.00577 | 2.12E-06 | 22.55011673 |
|  | rs6471209 | G | A | -0.0264 | 0.00569 | 3.41E-06 | 21.52695564 |
|  | rs4013190 | A | G | -0.03 | 0.0061 | 8.50E-07 | 24.18700723 |
|  | rs12686676 | G | A | -0.0265 | 0.00571 | 3.35E-06 | 21.53866722 |
|  | rs10159545 | G | C | 0.0274 | 0.00592 | 3.78E-06 | 21.42185164 |
|  | rs10762103 | A | G | -0.032 | 0.00699 | 4.60E-06 | 20.95776181 |
|  | rs3781295 | A | G | -0.0313 | 0.00581 | 7.35E-08 | 29.02255917 |
|  | rs7078576 | C | T | -0.0302 | 0.00613 | 8.10E-07 | 24.27125493 |
|  | rs147662818 | T | C | 0.14 | 0.0261 | 8.31E-08 | 28.77228488 |
|  | rs11021839 | C | G | 0.0695 | 0.0145 | 1.66E-06 | 22.97380337 |
|  | rs4275621 | G | A | -0.0274 | 0.00584 | 2.76E-06 | 22.01277155 |
|  | rs559928 | C | T | 0.034 | 0.00722 | 2.42E-06 | 22.17597554 |
|  | rs2186874 | C | T | 0.042 | 0.00576 | 2.99E-13 | 53.16831647 |
|  | rs12370612 | G | A | 0.0283 | 0.00574 | 8.16E-07 | 24.30795263 |
|  | rs7984262 | T | C | 0.0293 | 0.00628 | 3.06E-06 | 21.76783557 |
|  | rs1369588 | G | A | 0.033 | 0.00704 | 2.84E-06 | 21.97262058 |
|  | rs281287 | G | A | 0.0298 | 0.00592 | 4.82E-07 | 25.33893805 |
|  | rs62007780 | T | G | -0.0267 | 0.00578 | 3.90E-06 | 21.33861073 |
|  | rs12441907 | A | C | -0.0457 | 0.00716 | 1.90E-10 | 40.73855782 |
|  | rs12448767 | G | A | -0.0333 | 0.00731 | 4.99E-06 | 20.75166788 |
|  | rs11648954 | T | A | -0.0506 | 0.0108 | 2.94E-06 | 21.95092459 |
|  | rs11078716 | T | G | -0.029 | 0.00579 | 5.78E-07 | 25.08638964 |
|  | rs12453920 | C | G | -0.0411 | 0.00891 | 3.86E-06 | 21.2778396 |
|  | rs6110373 | T | C | -0.0482 | 0.0103 | 3.03E-06 | 21.89872965 |
| Cigarettes per day | rs2072659 | G | C | -0.0305 | 0.00637 | 1.71E-06 | 83.73418126 |
|  | rs11719922 | C | G | 0.0183 | 0.00399 | 4.73E-06 | 76.83133019 |
|  | rs56382648 | A | G | -0.0215 | 0.00465 | 4.01E-06 | 78.08236846 |
|  | rs73193343 | A | T | 0.0252 | 0.00552 | 4.98E-06 | 76.12107104 |
|  | rs167760 | A | G | -0.0231 | 0.00467 | 7.32E-07 | 89.36596777 |
|  | rs112709602 | T | C | 0.0466 | 0.0101 | 3.72E-06 | 77.75190706 |
|  | rs215600 | A | G | -0.0249 | 0.00392 | 2.06E-10 | 147.3698102 |
|  | rs7814146 | A | C | 0.0195 | 0.004 | 9.92E-07 | 86.80229429 |
|  | rs58379124 | C | T | 0.0336 | 0.00439 | 2.06E-14 | 213.9594528 |
|  | rs7820068 | A | G | -0.0247 | 0.00485 | 3.67E-07 | 94.73105925 |
|  | rs13296360 | C | G | 0.0181 | 0.00376 | 1.57E-06 | 84.63761639 |
|  | rs11139219 | T | C | -0.0242 | 0.00467 | 2.31E-07 | 98.07965624 |
|  | rs1999066 | C | G | -0.0239 | 0.00472 | 4.20E-07 | 93.64697834 |
|  | rs67401159 | G | A | 0.0315 | 0.00652 | 1.35E-06 | 85.25264858 |
|  | rs3025383 | C | T | -0.0319 | 0.00471 | 1.19E-11 | 167.5411592 |
|  | rs4331004 | A | C | 0.0249 | 0.00523 | 1.92E-06 | 82.78988525 |
|  | rs2118359 | T | G | 0.0264 | 0.00461 | 1.03E-08 | 119.781009 |
|  | rs75494138 | T | C | 0.0388 | 0.00725 | 8.96E-08 | 104.6090847 |
|  | rs75007400 | C | T | 0.0794 | 0.0174 | 4.78E-06 | 76.05442241 |
|  | rs10736470 | A | G | 0.0189 | 0.00394 | 1.64E-06 | 84.04524139 |
|  | rs9788721 | T | C | -0.0902 | 0.00402 | 1.27E-111 | 1838.835727 |
|  | rs2010752 | C | T | 0.0379 | 0.00812 | 3.06E-06 | 79.56981084 |
|  | rs182317 | T | G | -0.0201 | 0.00402 | 5.70E-07 | 91.31076322 |
|  | rs1579233 | G | A | -0.0228 | 0.00379 | 1.94E-09 | 132.1822799 |
|  | rs2016968 | G | C | -0.0192 | 0.00381 | 4.71E-07 | 92.75438927 |
|  | rs34636412 | G | A | -0.0223 | 0.00476 | 2.71E-06 | 80.16370567 |
|  | rs55978930 | G | A | 0.0193 | 0.00414 | 2.97E-06 | 79.37722279 |
|  | rs56057442 | T | C | -0.0277 | 0.00597 | 3.50E-06 | 78.63082639 |
|  | rs2860511 | A | G | 0.0208 | 0.00425 | 1.03E-06 | 87.4844315 |
|  | rs34406232 | A | C | -0.0658 | 0.0112 | 4.20E-09 | 126.0659225 |
|  | rs56113850 | C | T | 0.0402 | 0.00383 | 8.58E-26 | 402.3801261 |
|  | rs6141311 | T | C | 0.0201 | 0.00381 | 1.35E-07 | 101.6539193 |
|  | rs2273500 | C | T | 0.0345 | 0.00532 | 8.58E-11 | 153.6020068 |
|  | rs117087064 | C | T | 0.0916 | 0.0196 | 3.04E-06 | 79.77388978 |
| Rotator cuff tears | rs4725069 | C | T | -0.1260 | 0.0215 | 6.56E-09 | 34.17436041 |
|  | rs117822519 | A | G | 0.2783 | 0.0545 | 3.98E-07 | 26.09827967 |
|  | rs227752 | C | T | -0.1072 | 0.0211 | 4.77E-07 | 25.74754862 |
|  | rs138677057 | A | T | -0.4068 | 0.0808 | 5.88E-07 | 25.33100152 |
|  | rs17221337 | T | G | -0.1861 | 0.0377 | 9.82E-07 | 24.32818873 |
|  | rs1580278 | A | C | 0.0947 | 0.0192 | 9.84E-07 | 24.31674569 |
|  | rs114050254 | A | G | 0.6335 | 0.1292 | 1.14E-06 | 24.04505463 |
|  | rs10151263 | T | C | -0.1663 | 0.0341 | 1.26E-06 | 23.84052599 |
|  | rs200473311 | T | C | 0.1860 | 0.0381 | 1.28E-06 | 23.81911863 |
|  | rs75466231 | G | A | -0.1282 | 0.0267 | 1.92E-06 | 23.02391874 |
|  | rs188332471 | G | A | -0.1960 | 0.0410 | 2.12E-06 | 22.82448199 |
|  | rs62340622 | C | T | -0.4618 | 0.0976 | 2.67E-06 | 22.37385677 |
|  | rs73037953 | A | G | 0.1258 | 0.0267 | 2.85E-06 | 22.25099779 |
|  | rs4912314 | T | C | 0.1082 | 0.0231 | 3.29E-06 | 21.96814768 |
|  | rs192126774 | T | A | -0.4087 | 0.0880 | 4.07E-06 | 21.55610823 |
|  | rs9323805 | T | G | -0.0884 | 0.0192 | 4.81E-06 | 21.23590414 |
