# Supplementary material for: Mendelian randomization analysis does not reveal a causal influence of smoking on rotator cuff tears
Source: Medicine (Baltimore). 2025 Oct 10;104(41):e45212. doi: 10.1097/MD.0000000000045212 (PMC12517924; doi:10.1097/MD.0000000000045212)
Supplement: Supplementary file 2 [file medi-104-e45212-s002.docx]

**
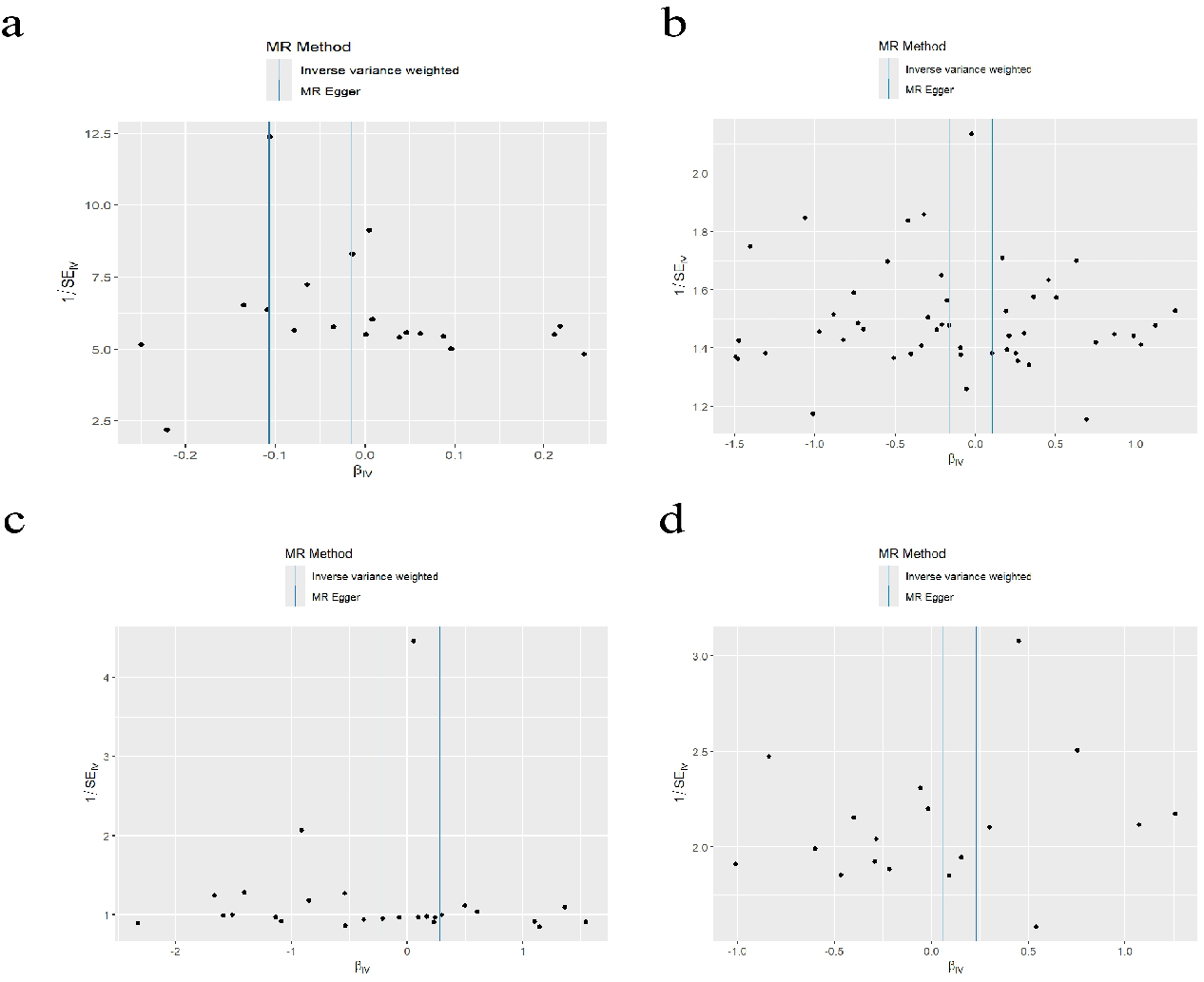
**

**Supplementary Figure S1.** The funnel plot of the effect of smoking on rotator cuff tears

1. The funnel plot of the effect of smoking on rotator cuff tears
2. The funnel plot of the effect of smoking initiation on rotator cuff tears
3. The funnel plot of the effect of cigarettes per day on rotator cuff tears
4. The funnel plot of the effect of smoking cessation on rotator cuff tears

| 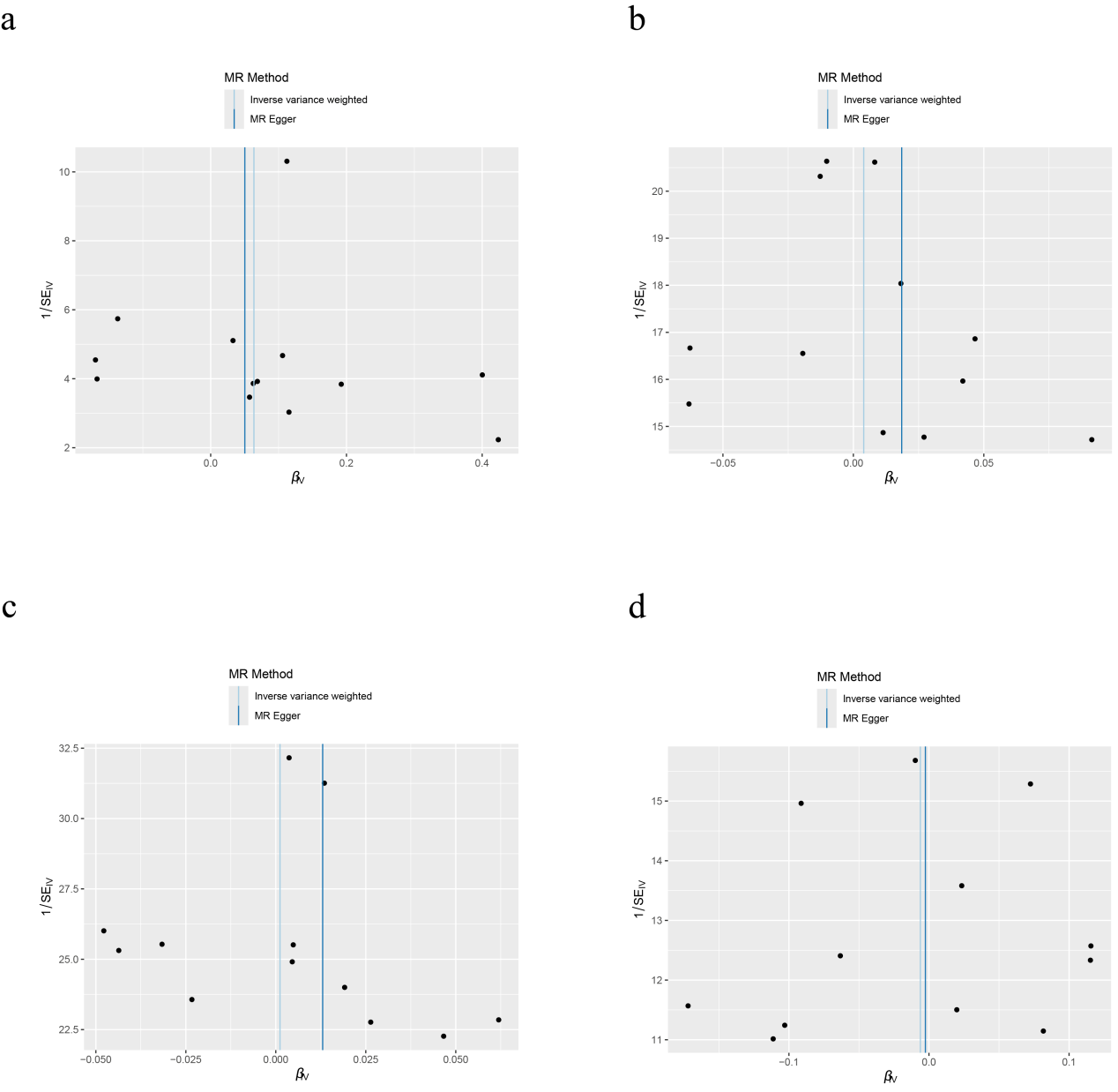 |
| --- |
| **Supplementary Figure S2.** The funnel plot of the effect of rotator cuff tears on smoking   1. The funnel plot of the effect of rotator cuff tears on smoking 2. The funnel plot of the effect of rotator cuff tears on smoking initiation 3. The funnel plot of the effect of rotator cuff tears on cigarettes per day 4. The funnel plot of the effect of rotator cuff tears on smoking cessation |
